# Supplementary material for: Comparison of Post-Thaw Motility and In Vitro Fertility Between Ejaculated and Epididymal Semen, and Seminal cfDNA Characterization in Pantaneiro Bulls
Source: Biology (Basel). 2025 Apr 25;14(5):465. doi: 10.3390/biology14050465 (PMC12108620; doi:10.3390/biology14050465)
Supplement: Supplementary file 1 [file biology-14-00465-s001.zip › biology-3562343-supplementary.pdf]

Table S1. Descriptive analysis presenting the median values of sperm motility and kinematics parameters in ejaculate and epididymal samples post-thawing (0h), alongside cfDNA concentration analysis in ejaculated samples.

	Ejaculate					*Epididymis*				
Bull ID	A	B	C	D*	E*	A	B	C	D*	E*
cfDNA (ng/ml)	31.1	43.1	50.9	21.6	11.4	-	-	-	-	-
Mot (%)	62.3	37.3	28.3	17.7	16.3	92.7	90.0	79.7	93.7	80.0
Prog (%)	30.0	14.7	12.0	7.3	8.0	74.7	71.0	49.7	77.3	63.0
VAP (µm/seg)	91.9	69.7	71.3	63.0	67.5	106.0	91.3	76.5	116.7	96.0
VSL (µm/seg)	64.4	45.4	51.5	46.1	52.5	88.3	74.4	56.1	96.0	77.4
VCL (µm/seg)	31.9	30.6	30.6	40.1	36.0	37.3	35.4	31.0	35.7	34.3
STR (%)	69.7	67.3	70.7	75.7	77.0	82.0	80.7	73.0	80.7	79.0
Lin (%)	37.0	33.7	36.3	37.3	37.3	48.7	46.7	37.7	47.3	43.0
Used for IVP	Yes	Yes	Yes	No	No	Yes	Yes	Yes	No	No

The asterisk (*) indicates bulls with Ejaculate post-thaw sperm motility below 20%, which were therefore not used for IVP, in both groups (Ejaculate and Epididymis).
